# Bronchiectasis and hoarseness of voice in takayasu arteritis: a rare presentation

**DOI:** 10.1186/1756-0500-5-447

**Published:** 2012-08-20

**Authors:** Gamage ND Perera, Anusha C Jayasinghe, Lalindra D Dias, Aruna Kulatunga

**Affiliations:** 1 National Hospital of Sri Lanka, Colombo, Sri Lanka

**Keywords:** Takayasu arteritis, Bronchiectasis, Recurrent laryngeal nerve palsy, Pericardial effusion

## Abstract

**Background:**

Takayasu arteritis is a large vessel vasculitis occurring in young females. We report a rare presentation of Takayasu arteritis in a Sri Lankan woman. She presented with bronchiectasis and left recurrent laryngeal nerve palsy prior to the onset of vascular symptoms. This case illustrates an atypical presentation of this disease and the diagnostic dilemma that the physician may be faced with.

**Case presentation:**

A 39-year-old woman presented with chronic cough, haemoptysis and hoarseness of voice. She had left recurrent laryngeal nerve palsy and high inflammatory markers on investigation. CT thorax revealed aortic wall thickening and traction bronchiectasis. 2 D echocardiogram revealed grade 1 aortic regurgitation compatible with aortitis. She did not have weak peripheral pulses or a blood pressure discrepancy and did not meet American College of Rheumatology (ACR) criteria for diagnosis of Takayasu arteritis at this stage. Tuberculosis, syphilis and sarcoidosis was excluded. While awaiting angiography, she developed left arm claudication and a pericardial effusion. Angiography revealed evidence of Takayasu arteritis and absence of flow in the left subclavian artery. Takayasu arteritis was diagnosed at this stage after a period of eight months from the onset of initial symptoms. She is currently on prednisolone, azathioprine and aspirin.

**Conclusion:**

Bronchiectasis and recurrent laryngeal nerve palsy is a rare presentation of Takayasu arteritis. Atypical presentations can occur in Takayasu arteritis prior to the onset of vascular symptoms. Elevation of inflammatory markers are an early finding. A high degree of suspicion is needed to identify these patients in the early course of the disease.

## Background

Takayasu arteritis (TA) is a granulomatous panarteritis of large vessels with a predilection for the aorta and its branches, pulmonary and the coronary arteries. It is commonly seen in Asian females of less than 40 years. Most patients develop limb claudication, hypertension, symptoms of organ ischaemia due to vascular narrowing, thrombosis or aneurismal dilatation of large arteries. These characteristic symptoms may be preceded by a pre pulseless systemic phase of constitutional symptoms. These symptoms are non specific and closely resemble many diseases causing diagnostic confusion. It is quite rare for Takayasu arteritis to present with bronchiectasis and recurrent laryngeal nerve palsy in the absence of vascular symptoms. We report such a case of Takayasu arteritis in a Sri Lankan woman. She developed the classical symptoms of limb ischaemia and a pericardial effusion, eight months after the initial presentation. Literature revealed one case of bronchiectasis in a patient with a transient ischaemic attack due to Takayasu arteritis.

This case illustrates the diagnostic dilemma faced by the treating physician when confronted with these atypical presentations and the need to consider this disabling disease as a possibility.

## Case history

A 39-year-old previously healthy woman presented to the local hospital with chronic cough, haemoptysis and progressive hoarseness of voice for 3 months. She did not have fever or a past or contact history of tuberculosis. But she had fatigability, loss of appetite and loss of weight. Preliminary investigations revealed markedly elevated inflammatory markers (ESR: 140 mm 1st hr, C reactive protein: 48 mg/L), thrombocytosis and anaemia of chronic disease. Chest radiograph was unremarkable except for haziness around the aortic knuckle. There was no evidence of pulmonary tuberculosis or a lung mass.

**Figure 1:**
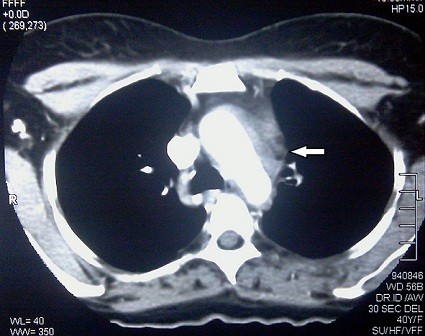
Contrast CT thorax demonstrating the thickening of aortic wall. (White arrow).

**Figure 2:**
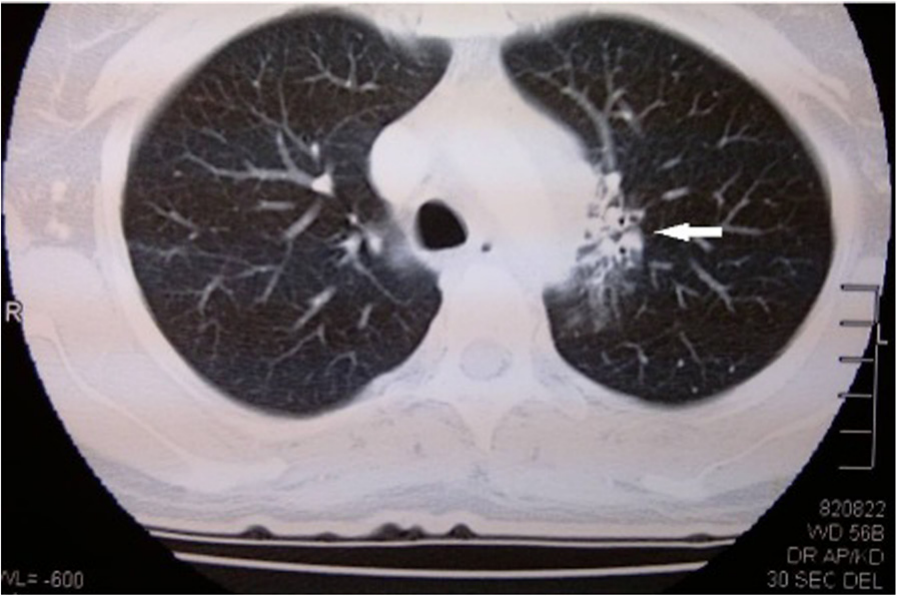
Contrast enhanced CT thorax of our patient illustrating traction bronchiectasis. (Arrow).

Indirect laryngoscopy revealed left sided vocal cord paralysis. CT thorax revealed diffuse thickening of the aortic arch involving the left recurrent laryngeal nerve. There was associated traction bronchiectasis accounting for her symptoms of cough and haemoptysis. (Figure 1 and Figure 2).

These findings raised the possibility of aortitis and a 2 D echocardiogram was performed. Echocardiogram revealed patchy aortitis involving the aortic arch with grade 1 aortic regurgitation. She denied claudication of limbs. Examination of the peripheral pulses was normal. There was no blood pressure discrepancy in the upper limbs. Patient did not meet criteria for a diagnosis of Takayasu arteritis at this stage. Sputum studies for acid fast bacilli and Mantoux test for tuberculosis was negative. Treponema Pallidum Haemagglutination assay was negative. Sarcoidosis was also considered and excluded. Antinuclear antibody was negative. Aortic arch angiography was scheduled.

**Figure 3:**
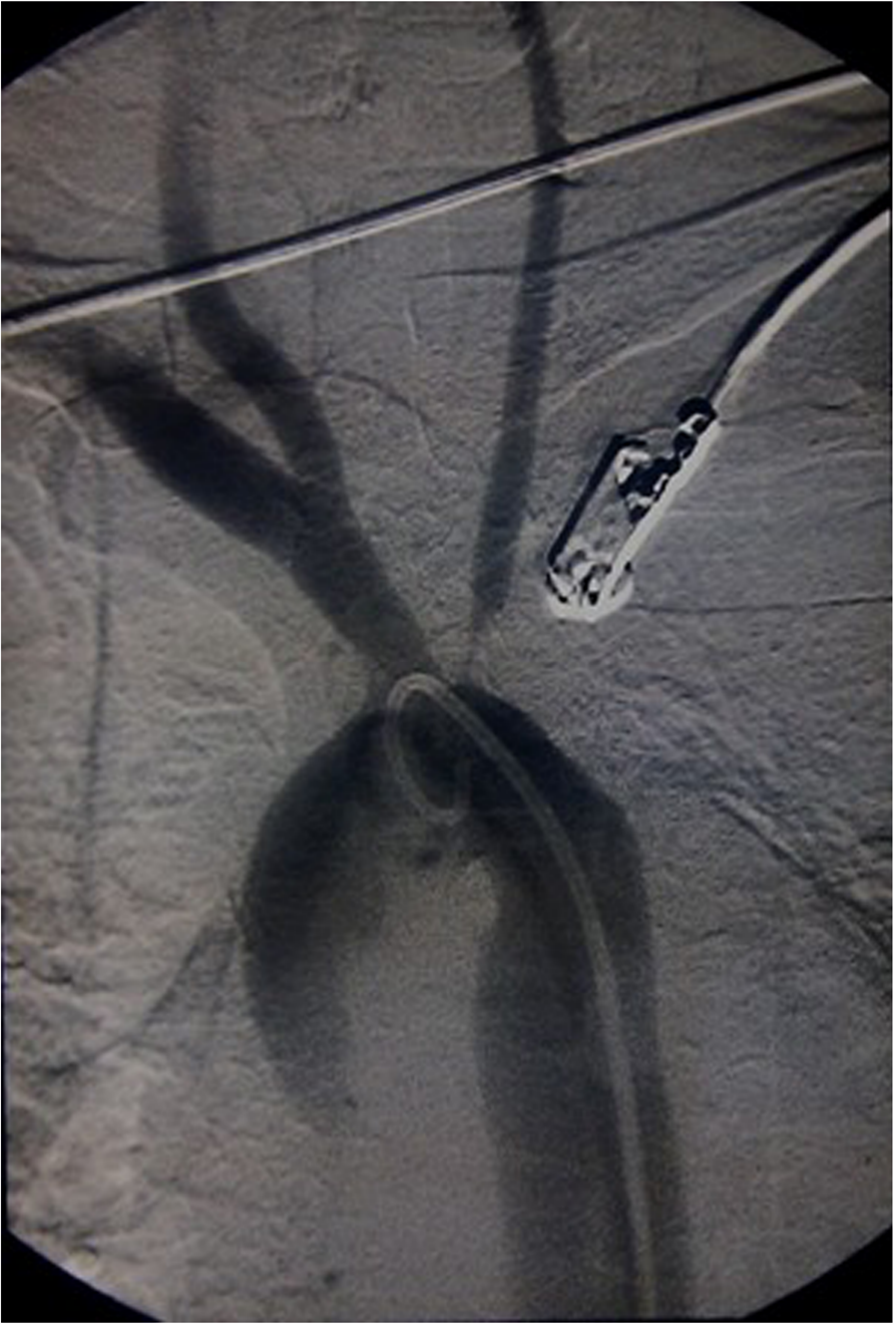
Aortic angiogram demonstrating absent flow in the left subclavian artery withnarrowing of the left common carotid artery.

While awaiting angiography, she developed dyspnoea and left arm claudication. There was evidence of a pericardial effusion on repeat echocardiography. Left radial pulse was absent with an unrecordable blood pressure in the left arm. We started oral prednisolone at this stage as the clinical features were highly suggestive of Takayasu arteritis. Aortic arch angiogram revealed absence of flow in the left subclavian artery and narrowing of the left common carotid artery (Figure 3). Aortic lumen was narrowed with patent renal arteries. She developed type 2 diabetes during this period. Patient had a dramatic improvement for steroids with appearance of the left radial pulse within one week.

Takayasu arteritis was diagnosed according to ACR criteria, 8 months after the onset of her initial symptoms and she was started on oral prednisolone, azathioprine, low dose aspirin and insulin for diabetes. Her constitutional symptoms disappeared with the resolution of inflammatory markers. But she continues to have left arm claudication and hoarseness of voice despite treatment with immunosuppressive agents. Repeat CT angiogram was performed 3 months following steroid treatment. There was no progression of vascular inflammation nor radiographic resolution in the repeat imaging.

## Discussion

Takayasu arteritis, first described by Dr Mikito Takayasu, an ophthalmologist in 1908 [1], is a granulomatous panarteritis of medium and large vessels affecting predominantly the aorta and its branches, coronary arteries and pulmonary arteries [2]. It is also known as aortic arch syndrome or pulseless disease and is thought to be of autoimmune origin [2,3]. It is ten times more common in females than males of 10–40 years of age [3]. Takayasu arteritis is a rare disease occurring in 6 per 1000 population worldwide with a higher incidence in Japan and in the Asian continent [3]. Prevalence in Sri Lanka is yet unknown.

TA is divided into an initial pre pulseless systemic phase and a later occlusive phase [3]. Patients present with constitutional symptoms, fever, and arthralgia during the pre pulseless stage and limb claudication, neurological symptoms, hypertension due to arterial ischaemia during the occlusive stage of the disease. These symptoms are non specific and closely resemble many inflammatory conditions (giant cell arteritis, sarcoidosis, Behcet's disease) and infections (tuberculosis, syphilis) [4]. Tuberculous aortitis is an important differential diagnosis in our country, Sri Lanka where infections due to Mycobacterium tuberculosis is still prevalent. About 9000 new cases of tuberculosis are notified every year in Sri Lanka [5] and though uncommon, tuberculosis can present with aortic arch involvement [6]. Presence of bronchiectasis further raised suspicion in our case of a possible tuberculous aetiology.

We report the case of a patient with an unusual initial presentation. She had left recurrent laryngeal nerve palsy and bronchiectasis. Literature search revealed one case of upper lobe bronchiectasis with TA [3]. A high degree of suspicion is needed to identify these atypical presentations of Takayasu arteritis. It is important to identify this disabling disease early and to initiate treatment. Our case further illustrates the possibility of having a long systemic phase before the onset of definite vascular symptoms. Pericardial involvement is also a rare manifestation of TA, reported by only a few authors [7].

Current diagnostic criteria was established in 1990 by the American college of Rheumatology [8]. It is sensitive in diagnosing TA in the occlusive stage of the disease but has doubtful value in the early stages of the illness. There are no diagnostic serological tests identified in Takayasu arteritis. But thrombocytosis is an early discriminatory feature [9]. Imaging modalities include angiography, CT, MRA and 18 F-fluorodeoxyglucose positron emission tomography (18 F-FDG-PET). Angiography was considered the gold standard imaging study to evaluate the disease extent and was used to identify the type of vascular involvement. But recent studies suggest that noninvasive tests are equally effective and probably superior in diagnosing Takayasu arteritis [10,11]. Vessel wall oedema and thickening are better indicators of early vascular involvement than narrowing.

Establishing a diagnosis of Takayasu arteritis is therefore challenging. It is further difficult when the presentation is in the initial phase without clinically evident arterial occlusion as in our patient.

Treatment of TA is as challenging as diagnosing the disease. Current data support the use of steroids and immunosuppressive therapy (Azathioprine, methotrexate, cyclophosphamide) tailored to the individual patient [12]. Surgical revascularization techniques have been used with success for critical vessel narrowing [12].

Our patient responded to a combination of prednisolone and azathioprine. Her left radial pulse reappeared. Though there was a clinical improvement and the radiological progression was halted, repeat imaging failed to show significant radiological resolution. Currently her disease activity is monitored by the inflammatory markers and improvement in clinical symptoms.

## Conclusion

Bronchiectasis and recurrent laryngeal nerve palsy is a rare presentation of Takayasu arteritis. It is difficult to identify patients with these atypical presentations and to differentiate from diseases with similar manifestations. Thrombocytosis and elevation of inflammatory markers are early findings which should prompt the clinician to search for evidence of Takayasu arteritis with appropriate imaging modalities.

### Consent

Written informed consent was obtained from the patient for publication of this case report and any accompanying images. A copy of the written consent is available for review by the series editor of this journal.

## Abbreviations

TA: Takayasu arteritis; ACR: American College of Rheumatology.

## Competing interests

The author(s) declare that they have no competing interests.

## Authors’ contribution

GP is the first author and wrote the manuscript. DJ and LD was involved in patient management and in writing the article. AK was responsible for overall management of the patient and supervised the writing critically. All the authors have made significant contributions for this manuscript. All authors read and approved the final manuscript.
